# Intrabody Cage Augmentation in Kümmell Disease and Osteoporotic Burst Fractures: Technical Insights and Narrative Review of Current Evidence

**DOI:** 10.3390/jcm15103790

**Published:** 2026-05-14

**Authors:** Sun Woo Jang, Junseok W. Hur, Younggyu Oh, Sungjae An, Jin Hoon Park, Subum Lee

**Affiliations:** 1 Department of Neurosurgery, Gangneung Asan Hospital, University of Ulsan College of Medicine, Gangneung 25440, Republic of Korea; sunwoo0118@naver.com; 2 Department of Neurosurgery, Korea University Anam Hospital, Korea University College of Medicine, Seoul 02841, Republic of Korea; hurjune@korea.ac.kr (J.W.H.); oyk1223@hanmail.net (Y.O.); annuguri@naver.com (S.A.); 3 Department of Neurosurgery, Asan Medical Center, University of Ulsan College of Medicine, Seoul 05505, Republic of Korea; spinejhpark@naver.com

**Keywords:** Kümmell disease, osteoporosis, intrabody cage augmentation, anterior column reconstruction, minimally invasive spine surgery, 3D-printed titanium cage

## Abstract

Intrabody cage augmentation has emerged as a minimally invasive technique for anterior column reconstruction in Kümmell disease and osteoporotic burst fractures. These osteoporotic conditions lead to progressive vertebral collapse, kyphosis, and instability. While cement augmentation provides rapid pain relief, it often fails to reliably restore sagittal balance or ensure biological integration in advanced stages of collapse. Although conventional anterior corpectomy with long-segment posterior fusion can achieve satisfactory deformity correction, these procedures are associated with substantial surgical morbidity. In contrast, screw fixation alone often fails to withstand anterior loading, resulting in loss of correction or hardware failure. By adapting standard interbody devices for off-label intravertebral use, this technique utilizes the intravertebral cleft as a natural cavity to restore vertebral height and sagittal alignment while preserving adjacent intervertebral discs and reducing stress on posterior instrumentation. The surgical technique involves transpedicular access, meticulous curettage of necrotic tissue, and insertion of a cage packed with osteoinductive material. This approach minimizes surgical trauma and operative time compared with conventional corpectomy procedures. Reported outcomes from retrospective series suggest promising pain relief, maintenance of correction, and low complication rates. Collectively, current evidence suggests that intrabody cage augmentation may serve as a potential, less invasive surgical option, acting as an intermediate approach between cement augmentation and corpectomy. However, as the existing evidence remains preliminary, high-quality prospective comparative studies are required to establish definitive indications and long-term efficacy.

## 1. Introduction

Kümmell disease, also known as delayed post-traumatic vertebral collapse with an intravertebral vacuum phenomenon, represents a distinct sequela of osteoporotic vertebral compression fractures (OVCFs) [1]. It primarily affects older adults with fragile bone quality and manifests as progressive kyphotic deformity, intravertebral instability, and, in advanced stages, neurological deficits [2]. The underlying pathogenesis is attributed to ischemic necrosis of the vertebral body, which leads to pseudarthrosis formation between the superior and inferior endplates and the characteristic intravertebral cleft (IVC) sign [2,3].

Management of Kümmell disease remains challenging. Affected patients are frequently frail older adults burdened by severe systemic osteoporosis, multiple cardiopulmonary comorbidities, and generally poor nutritional status. These underlying vulnerabilities significantly increase the risks of intraoperative bleeding, implant failure, and perioperative complications during extensive reconstructive surgeries. Consequently, conservative management, cement augmentation, or posterior screw fixation alone often fails to withstand ventral compressive loading. In patients with severe osteoporosis, pedicle screw fixation can not provide adequate anterior column support to maintain sagittal balance. Although anterior corpectomy and reconstruction enable direct decompression and effective deformity correction, these procedures are associated with prolonged operative time, increased blood loss, and elevated cardiopulmonary risks, particularly in older adults [4,5].

Notably, the IVC, located between relatively preserved endplates, provides a potential space for anterior column support. Ischemic necrosis within the vertebral body typically leads to a pseudarthrosis gap bounded by sclerotic endplates. Because these endplates remain relatively intact compared to the collapsed cancellous bone, they can functionally act like the native vertebral endplates in standard interbody fusion. This allows the endplates to securely anchor the intrabody cage, providing structural support without the need to sacrifice the adjacent discs. This anatomical feature provides the surgical adaptation of standard interbody devices for off-label intravertebral use, enabling anterior reconstruction through a posterior corridor while minimizing surgical morbidity [6].

This review summarizes the evolution of this technical approach and evaluates its biomechanical rationale and clinical efficacy as an emerging intermediate option in the treatment of osteoporotic burst fractures and Kümmell disease. Specifically, this study aims to identify distinct surgical indications—particularly differentiating between Kümmell disease stages—and critically assess the safety and long-term durability of intrabody cage augmentation compared with conventional cement augmentation and anterior corpectomy.

## 2. Methods

### 2.1. Literature Search Strategy

To ensure the transparency and reproducibility of this narrative review, a comprehensive literature search was conducted across three electronic databases: PubMed, Scopus, and Web of Science. The search encompassed literature published from January 2000 to April 2026. The Boolean search strategy utilized the following keywords and combinations: (“Kümmell disease” OR “osteoporotic vertebral compression fracture” OR “osteoporotic burst fracture”) AND (“intrabody cage” OR “anterior column reconstruction” OR “transpedicular cage insertion”).

### 2.2. Inclusion and Exclusion Criteria

Studies were selected based on their direct relevance to intrabody cage augmentation for osteoporotic spinal conditions. Inclusion criteria comprised clinical case series, technical notes, and biomechanical or finite-element analysis studies evaluating transpedicular anterior column reconstruction using an intrabody cage. Articles published in languages other than English, abstract-only publications, and studies lacking sufficient surgical detail were excluded. Additionally, the reference lists of all included articles were manually cross-referenced to identify any further relevant studies.

## 3. Technical Refinement of Intravertebral Cage Placement: From Early Adaptations to Minimally Invasive Approaches

The surgical adaptation of interbody devices for intrabody support has been gradually refined over the past two decades, with applications in both Kümmell disease and osteoporotic burst fractures (Table 1). The earliest report by Li et al. introduced the use of a transpedicle body augmenter (TpBA) as anterior column support in stage III Kümmell disease with cord compression [7]. In a retrospective cohort of 21 patients, this technique combined manual reduction with short-segment fixation and achieved significant kyphosis correction and vertebral height restoration, with low rates of implant failure during a mean follow-up of 48 months. This study established the foundation for posterior-only approaches that provide anterior support without extensive anterior surgery.

Subsequently, Lee et al. reported the first use of transpedicular intrabody cage insertion with posterior stabilization in two patients with Kümmell disease [8]. Both cases achieved satisfactory pain relief and maintenance of vertebral body height on follow-up imaging, demonstrating the technical feasibility of cage-based anterior support through a posterior approach.

Building on these findings, Lee and Song presented a series of 10 patients with severely collapsed Kümmell disease and neurological deficits, treated with posterior-only intravertebral cage augmentation [9]. At more than 2 years of follow-up, patients demonstrated substantial improvements in pain, ambulatory function, and radiographic parameters, including vertebral height and kyphotic angle. This study highlighted that intravertebral cage augmentation could serve as a valuable alternative to combined anterior–posterior surgery in frail patients with osteoporosis.

During the same period, Chen et al. proposed a modified intrabody cage technique for stage III Kümmell disease [10]. Their technical note highlighted the potential of a less invasive cage insertion method with posterior stabilization to achieve acceptable deformity correction and fusion in advanced cases, providing an alternative to more invasive osteotomy procedures. Expanding the application beyond Kümmell disease, Chen et al. also investigated minimally invasive decompression with intracorporeal bone grafting and temporary short-segment fixation for thoracolumbar burst fractures with neurological deficits [11]. In a cohort of 84 patients, this approach achieved effective decompression, restoration of anterior height, and kyphosis correction, which were maintained after implant removal. Although no cage was used, the study reinforced the principle that anterior column reconstruction within the vertebral body is critical for long-term stability.

Recent developments have focused on technical refinements and minimally invasive adaptations. Park et al. described a percutaneous transpedicular intracorporeal cage grafting (PTICG) technique for Kümmell disease [12]. This method minimized disruption of paraspinal musculature while allowing anterior column reconstruction, suggesting the potential of percutaneous cage insertion as an intermediate approach between vertebroplasty and open surgery.

Recent advancements feature the use of expandable cages, which optimize transpedicular insertion. As demonstrated by Song et al. in a six-case series, these expandable devices navigate narrow pedicles more safely than static cages and expand in situ [13]. This mechanism allows for robust vertebral height restoration while significantly mitigating the risk of iatrogenic endplate injury. Early outcomes demonstrated durable maintenance of correction and substantial pain improvement.

Finally, Bae et al. presented the Minimally Invasive Surgery Transpedicular Intrabody Cage (MISTIC) technique in a series of 20 patients with Kümmell disease [14]. The procedure achieved substantial correction of segmental kyphosis, restoration of vertebral height, and a 100% fusion rate at 1 year, with sustained improvements in pain and functional outcomes. This approach represents the culmination of two decades of progress, integrating intrabody support principles with minimally invasive spinal surgery.

In summary, the reviewed literature demonstrates a technical shift: from early static cage augmentation and TpBA methods to modified and posterior-only techniques, and more recently, to expandable and minimally invasive applications. Collectively, these studies underscore the expanding role of intrabody cage insertion as a viable and potential surgical strategy for the management of osteoporotic burst fractures and Kümmell disease.

## 4. Pathophysiological and Biomechanical Rationale for Intrabody Cage Insertion

The rationale for intrabody cage insertion in Kümmell disease and osteoporotic burst fractures derives from both the distinctive pathological substrate and the biomechanical demands of these conditions. Unlike acute OVCFs, Kümmell disease results from ischemic necrosis of the vertebral body, leading to the formation of an IVC. This cleft functions as a pseudarthrosis, permitting abnormal micromotion, progressive instability, and kyphotic deformity. Conventional cement augmentation may provide transient pain relief; however, it fails to reconstruct the anterior column or reliably restore sagittal balance, particularly in advanced stages of collapse [7,15].

A key anatomical feature of Kümmell disease is that the intervertebral discs are often preserved despite progressive vertebral body collapse. Because the necrotic cavity is localized between relatively intact endplates, surgical removal of the intervertebral disc and formal interbody fusion are unnecessary. The cleft itself can serve as a receptacle for structural support. Inserting a cage into this cavity allows restoration of vertebral height, reconstruction of the anterior column, and maintenance of physiological load-sharing across preserved discs—while avoiding the morbidity associated with anterior approaches [8,10].

Biomechanically, posterior instrumentation alone is frequently insufficient to counteract ventral compressive loads in severely osteoporotic bone. Reconstruction of the anterior column with an intrabody cage fundamentally alters load-sharing. Finite-element analyses and cadaveric studies demonstrate that when a structural cage is seated between the endplates, axial forces are transmitted through the cage rather than being concentrated on posterior screws and rods. For instance, cadaveric models quantifying cage force under axial loading reveal that an anteriorly placed cage bears a substantial proportion (up to 50%) of the total compressive load, demonstrating effective anterior–posterior load-sharing [16,17]. Furthermore, computational models demonstrate that optimizing posterior instrumentation—such as placing additional pedicle screws at the fracture level—can further reduce peak mechanical stress on the implants by approximately 30% [18]. The combination of anterior column support and optimized posterior fixation directly correlates with a lower risk of hardware fatigue, decreased screw loosening, and a minimized incidence of pull-out at the upper and lower instrumented vertebrae (UIV/LIV), thereby maximizing overall construct longevity [16,18,19,20].

These physical findings are further supported by finite-element modeling (FEM). Although FEM inherently oversimplifies the complex biological behaviors of living osteoporotic bone, it provides valuable controlled analyses of mechanical variables. These computational models indicate that constructs spanning the collapsed body with an anterior device demonstrate higher stiffness and reduced implant stress compared with posterior pedicle screw fixation alone [18]. Similarly, deformity correction models indicate that anterior column reconstruction reduces rod stress relative to posterior osteotomy-only approaches [21]. By corroborating these computational models with actual cadaveric pressure dynamics, the biomechanical rationale for anterior support—specifically, lowering the risk of hardware fatigue and recurrent kyphosis—becomes highly reliable.

Recent implant innovations further enhance these biomechanical advantages. Expandable cages permit controlled height restoration through narrow pedicles while minimizing endplate injury [10,13], and additive-manufactured porous titanium cages provide superior osteoconduction and efficient stress conduction compared with polyetheretherketone (PEEK) devices [14,22]. These advances not only promote bone healing potential but also reinforce the cage’s role as a true load-bearing anterior column substitute. In addition to mechanical stabilization, height restoration through cage expansion applies tension to the posterior wall and posterior longitudinal ligament (PLL), facilitating indirect reduction in retropulsed fragments, a mechanism known as ligamentotaxis. This mechanism explains why neurological improvement can occur even in the absence of direct anterior decompression [8].

## 5. Management Strategies for Osteoporotic Burst Fracture and Kümmell Disease

Surgical management of osteoporotic burst fractures and Kümmell disease requires careful evaluation of bone quality, the degree of vertebral collapse, neurological status, and patient comorbidities. In many older adults, initial conservative management, including bracing, analgesia, and anti-osteoporotic medication, may be effective [23,24,25]. However, progression to intractable pain, pseudarthrosis, progressive kyphotic deformity, or neurological compromise often necessitates surgical intervention [25,26,27].

## 6. Staging of Kümmell Disease and Treatment Implications

Kümmell disease is commonly classified into three stages, and treatment decisions are strongly stage-dependent [3]:

‒ Stage I: Characterized by an IVC without significant collapse or kyphosis. Patients typically present with back pain but no neurological deficits. Conservative management or minimally invasive cement augmentation (vertebroplasty, kyphoplasty) is usually sufficient.
‒ Stage II: Marked by progressive vertebral body collapse with intravertebral instability and localized kyphosis. Neurological compromise is typically absent or mild. Surgical stabilization with intrabody cage insertion is advantageous at this stage, as it restores vertebral height and sagittal alignment while minimizing morbidity.
‒ Stage III: Defined by severe collapse with retropulsion of bony fragments into the spinal canal and potential neurological deficits. Posterior decompression (laminectomy), followed by intrabody cage reconstruction and posterior fixation, is recommended to relieve neural compression.

## 7. Comparative Surgical Options

As summarized in Table 2, a spectrum of treatment strategies exists, ranging from cement augmentation techniques such as vertebroplasty and kyphoplasty to more extensive procedures including posterior screw fixation, anterior corpectomy with posterior screw fixation, and intrabody cage augmentation. Each modality has inherent advantages and limitations. Cement augmentation provides rapid pain relief in the absence of neurological deficits; however, it is insufficient for advanced collapse or instability [28,29,30]. Furthermore, in the context of poor bone quality, injection of cement into the collapsed vertebra may increase stress transfer to adjacent segments, predisposing patients to subsequent adjacent vertebral fractures [31,32,33]. Conversely, anterior corpectomy with posterior screw fixation achieves robust correction but is associated with substantial operative morbidity in frail patients with osteoporosis [34,35,36].

Intrabody cage insertion has emerged as an intermediate option within this therapeutic continuum, providing anterior column support via a transpedicular approach. By restoring vertebral body height and maintaining sagittal alignment, the cage reduces mechanical stress on posterior instrumentation while avoiding the morbidity associated with anterior corpectomy procedures, including massive bleeding or thoracic root sacrifice [8,10,14]. Notably, this technique preserves the intervertebral disc, thereby substantially reducing operative time.

To optimize clinical utility, the indications and contraindications for intrabody cage augmentation must be strictly defined based on the stage of collapse and anatomical preservation. A structured decision algorithm is summarized as follows:

Indications:

‒ Stage II Kümmell Disease: Patients with progressive collapse and intravertebral instability but without significant canal compromise. This is the ideal indication for minimally invasive transpedicular cage augmentation without laminectomy.
‒ Select Stage III Kümmell Disease/Osteoporotic Burst Fractures: Patients with severe collapse and mild-to-moderate neurological deficits due to canal compromise. These cases require transpedicular cage augmentation combined with concomitant posterior decompression (laminectomy) to relieve neural pressure.

Absolute Contraindications:

‒ Severe endplate destruction: Complete loss of the superior or inferior endplates precluding stable cage anchorage or resulting in high risk of severe subsidence.
‒ Active spinal infection: Spondylodiscitis or epidural abscess.
‒ Multi-level severe adjacent segment degeneration: Cases requiring long-segment correction and fusion rather than short-segment stabilization.
‒ Severely compromised pedicle anatomy: Fractures or inherent deformities preventing safe transpedicular access.

In most cases, posterior fixation is combined with cage insertion, and cement augmentation of screws may further enhance stability.

**Figure 1:**
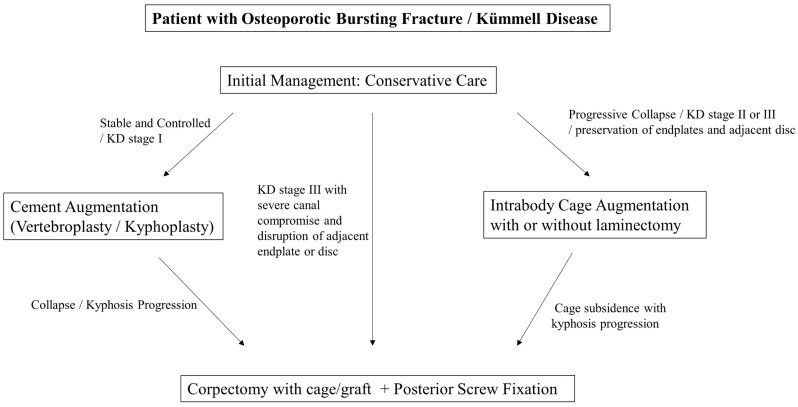
Treatment strategy for Kümmell disease. Conservative management is the initial approach. Stable and controlled cases, corresponding to Kümmell disease Stage I, are suitable for cement augmentation procedures, such as vertebroplasty or kyphoplasty. Progressive collapse or instability, corresponding to Kümmell disease Stage II or III with preservation of adjacent endplates and discs, can be effectively managed with intrabody cage augmentation via a transpedicular approach, with or without laminectomy. Kümmell disease Stage III with severe canal compromise or destruction of adjacent endplates and discs requires anterior column reconstruction using corpectomy with cage or graft, combined with posterior screw fixation. Progressive collapse or kyphotic progression secondary to cage subsidence after prior intrabody cage augmentation also warrants long-segment fusion.

Collectively, the choice of surgical strategy should balance the biological and biomechanical demands of the pathology with the patient’s tolerance for surgery. As illustrated in Figure 1, intrabody cage insertion occupies a unique position between cement-based augmentation and extensive reconstructive procedures, offering reliable stability with lower morbidity.

## 8. Surgical Technique

**Figure 2:**
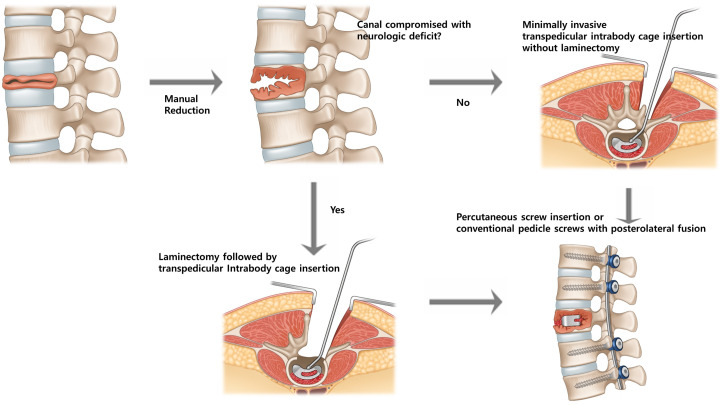
Surgical decision-making flow for intrabody cage insertion. Canal compromise and neurological status determine the operative strategy. When no significant canal compromise is present, a minimally invasive transpedicular intrabody cage insertion is performed without laminectomy, followed by percutaneous or conventional pedicle screw fixation. In cases with canal compromise and neurological deficit, laminectomy is first performed for decompression, followed by transpedicular intrabody cage insertion combined with short-segment fixation to reconstruct the anterior column.

**Figure 3:**
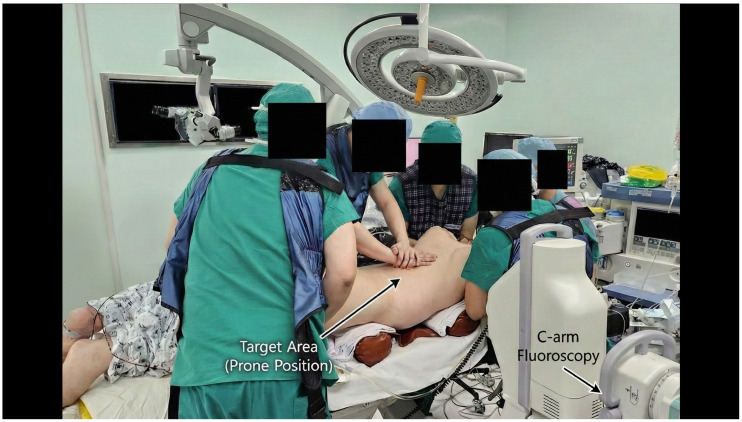
Intraoperative positioning for manual and gravity-assisted reduction. Kyphosis reduction is achieved by placing the patient prone on a Jackson table or by positioning two horizontal roll bars beneath the sternum and the ASIS, producing a three-point bending effect. This configuration facilitates manual reduction in the collapsed vertebra and gravity-assisted correction before transpedicular cage insertion.

Positional reduction was initially achieved using the Jackson Table, followed by manual reduction in the collapsed vertebra under fluoroscopic guidance. The subsequent surgical procedure was tailored to the presence of canal compromise and neurological deficit (Figure 2). In the absence of a Jackson Table, equivalent positioning can be achieved by placing two horizontal roll bars—one beneath the sternum and another beneath the anterior superior iliac spines (ASIS)—to create a three-point bending effect (Figure 3). This setup facilitates manual reduction and gravity-assisted kyphosis correction during the procedure.

In cases with mild canal compromise without neurological deficit, minimally invasive transpedicular intrabody cage insertion was performed without laminectomy. Under C-arm fluoroscopic guidance and through a tubular retractor, a working channel was created through the pedicle using a pedicle tapper and osteotome. Serial cage trialing was conducted, beginning with the smallest size (typically 8 mm) and increasing in 1 mm increments until the appropriate height was achieved, taking care to avoid endplate injury. To maximize osteoconduction between the titanium cage surface and host bone, necrotic tissue within the collapsed cavity was meticulously curetted from both the superior and inferior aspects, similar to endplate preparation in interbody fusion surgery. The cavity and/or cage was then packed with demineralized bone matrix (DBM) or BMP-2 to promote vertebral reconstruction. The appropriately sized intrabody cage was then inserted into the vertebral cleft to restore anterior column height and correct local kyphosis.

Posterior stabilization was achieved using pedicle screws. Percutaneous screws were generally preferred to minimize muscle dissection; however, in cases requiring posterior interlaminar fusion, conventional pedicle screws were placed through limited exposure while preserving the posterior tension band and paraspinal musculature.

In cases with severe canal compromise accompanied by neurological deficit, a laminectomy was first performed to decompress the spinal canal and relieve pressure on the cord or cauda equina. Through the same exposure, a transpedicular route was used for intrabody cage insertion to reconstruct the anterior column, followed by posterior pedicle screw fixation to maintain correction and prevent further collapse. Technical precautions included meticulous vertebral endplate preparation to prevent cage subsidence, careful cage size selection to avoid pedicle fracture during insertion, and optional use of cement-augmented screws in patients with severe osteoporosis.

## 9. Illustrative Case

To contextualize the broader evidence discussed in this review, the following case is presented as a highly representative clinical scenario. It illustrates a typical Stage III Kümmell disease patient presenting with severe kyphosis, instability, and neurological symptoms, directly reflecting the indications and successful outcomes—such as durable kyphosis correction and true biological reconstruction—reported in the cited literature.

A 60-year-old female sustained an osteoporotic vertebral fracture at T12 following a fall. She was initially managed conservatively; however, progressive collapse resulted in severe thoracolumbar kyphosis, a forward-leaning posture, and intractable back pain due to compensatory lumbar hyperextension. During ambulation, she also reported heaviness and cramping in both lower extremities, indicating surgical intervention.

**Figure 4:**
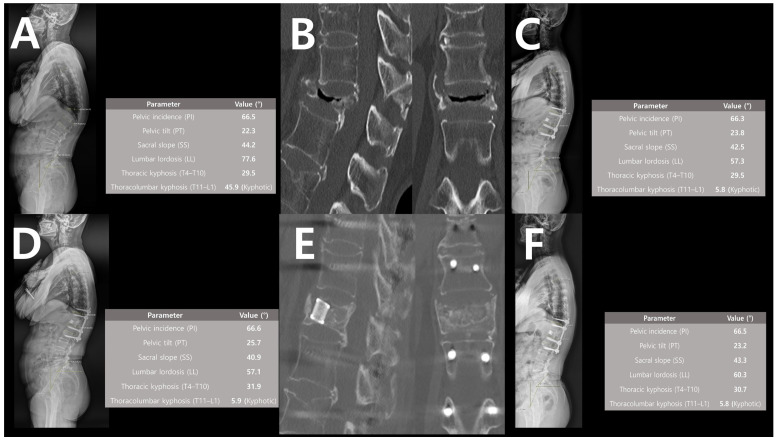
Illustrative case of intrabody cage augmentation for Kümmell disease at T12. (**A**) Preoperative radiograph showing severe thoracolumbar kyphosis (45.9°) and compensatory lumbar hyperlordosis. (**B**) Preoperative CT revealing a typical intravertebral vacuum cleft and severe vertebral collapse (Stage III). (**C**) 1-month postoperative radiograph demonstrating restored thoracolumbar alignment (5.8°). (**D**) 1-year postoperative radiograph showing maintained correction. (**E**) 1-year postoperative CT demonstrating complete osseous integration around the cage with preserved adjacent discs. (**F**) 2-year postoperative radiograph confirming stable alignment without hardware failure.

Preoperative standing whole-spine lateral radiograph demonstrated marked thoracolumbar kyphosis of 45.9° with compensatory lumbar hyperlordosis (Figure 4A). Computed tomography (CT) scans revealed a typical intravertebral vacuum cleft with vertebral body collapse consistent with Kümmell disease Stage III (Figure 4B). The patient underwent posterior fixation with transpedicular intrabody cage insertion at T12.

At 1 month postoperation, whole-spine lateral radiograph showed restoration of thoracolumbar alignment with kyphosis corrected to 5.8° and resolution of compensatory lumbar hyperlordosis (Figure 4C). At 1 year, postoperative radiographs showed that the correction angle and global sagittal balance were well maintained (Figure 4D). One-year follow-up CT images demonstrated complete vertebral body reconstruction via osteoconduction and osteointegration around the intrabody titanium cage, with preservation of both adjacent endplates and discs (Figure 4E). At 2-year follow-up, the correction remained stable, with maintained sagittal alignment and absence of hardware-related complications (Figure 4F).

## 10. Discussion

The management of Kümmell disease and osteoporotic burst fractures requires a careful balance between deformity correction, neural protection, and surgical morbidity in older adults with fragile bone. Intrabody cage augmentation provides an intermediate solution that combines the mechanical benefits of anterior reconstruction with the reduced morbidity of posterior-only approaches. By utilizing the IVC as a natural cavity for cage insertion, vertebral height can be restored, kyphosis corrected, and anterior load-sharing re-established without performing corpectomy. This approach offers several advantages for frail patients with osteoporosis, including minimized soft-tissue injury, shortened operative time, and reduced perioperative risk.

In Kümmell disease, a minimally invasive anterior reconstruction is essential for restoring stability. Previous studies have explored bone cement-augmented percutaneous short-segment fixation to provide anterior support while avoiding extensive fusion [37,38]. Although this approach achieves effective pain relief and partial restoration of vertebral height, it relies on polymethylmethacrylate (PMMA), which lacks biological osteointegration and may not sustain long-term height maintenance. The cement provides immediate mechanical reinforcement but does not promote natural bone healing within the collapsed vertebral body. Furthermore, PMMA constructs are susceptible to progressive osteolysis and stress concentration at the cement–bone interface, potentially resulting in delayed collapse or screw loosening in severely osteoporotic bone [39].

In contrast, intrabody cage augmentation using titanium or 3D-printed porous titanium cages filled with osteoinductive and osteoconductive materials, such as DBM, provides both immediate mechanical stability and robust biological reconstruction. The interconnected porous structure of 3D-printed titanium mimics the native trabecular architecture, providing an optimal scaffold for cellular adhesion and vascularization. When combined with DBM, this osteoconductive surface facilitates rapid bone ingrowth and remodeling within the vertebral body. This synergistic effect enables true osseous integration across the collapsed segment, ensuring the implant functions as a biologically active fusion mass rather than merely a static spacer. This process restores the physiological load-bearing capacity of the anterior column and enhances the long-term durability of the construct. Placement of the cage within the IVC restores vertebral height, indirectly decompresses the canal by reducing retropulsed bone fragments, corrects kyphotic deformity, and maintains sagittal alignment. Addressing chronic spinal pain is a central goal of these surgical strategies, as such debilitating pain significantly impairs both functional capacity and overall well-being [40]. Clinical series have consistently demonstrated that constructs incorporating intrabody cages preserve correction and reduce pain recurrence more effectively than cement augmentation and screw fixation [8,10].

A recurring debate is whether posterior fusion is necessary in addition to screw stabilization. Some reports indicate that percutaneous screw fixation alone may provide short-term stability in acute traumatic fractures [37,41]. However, in Kümmell disease and osteoporotic collapse with pseudarthrosis, screw-only stabilization often proves inadequate, being prone to kyphotic recurrence and hardware loosening [10]. When cage augmentation is performed, posterior fixation primarily provides temporary stability until bony reconstruction of the index vertebral body occurs; thus, percutaneous screw fixation without posterolateral fusion may suffice in selected cases, as it preserves motion segments and reduces surgical invasiveness. Nevertheless, fusion remains advisable in patients with severe osteoporosis, fragile endplates at risk of subsidence, or concomitant degeneration of adjacent segments, where additional reinforcement may prevent progressive deformity.

Recent innovations in expandable and 3D-printed cages further enhance reliability. Expandable cages allow controlled height restoration through narrow pedicles with minimal endplate violation. Furthermore, 3D-printed porous titanium cages provide a biologically active surface that promotes osteointegration and more physiological load transfer compared with conventional polyetheretherketone (PEEK) devices. While PEEK possesses an elastic modulus similar to cortical bone, its hydrophobic surface limits cellular adhesion. In contrast, the osteoconductive architecture of 3D-printed porous titanium is biologically superior, particularly in the poorly vascularized environment of Kümmell disease [13,14,22]. These innovations increase both mechanical reliability and biological healing potential of the construct, making cage-based augmentation more robust in fragile bone.

Another potential advantage of the intrabody cage technique is the potential to reduce overall fixation length. Prior studies demonstrate that inserting pedicle screws into the intact pedicle at the fractured vertebra can effectively reduce the number of instrumented segments [18,21]. Even when the cage is inserted through a single pedicle, a screw may be placed into the contralateral pedicle if its integrity is preserved, further supporting the feasibility of reduced fixation. Although conventional anterior corpectomy typically requires instrumentation at two levels above and below the fractured vertebra [42,43,44], intrabody cage studies provide no definitive recommendations regarding optimal fixation length, with reported constructs varying across series. In the absence of defined criteria, utilizing an intact pedicle at the fractured level for screw insertion represents a practical strategy to safely reduce fixation levels while maintaining adequate stability.

Beyond Kümmell disease, intrabody cage augmentation may be extended to other pathologies characterized by osteolytic vertebral destruction, such as metastatic or pathological burst fractures. In these cases, where anterior column support is required but anterior corpectomy carries prohibitive morbidity, transpedicular cage insertion provides a biomechanically effective, less-invasive alternative for spinal stabilization and ventral decompression.

From a future perspective, integration of endoscopic or percutaneous techniques may further refine this approach. Endoscope-assisted cage augmentation could permit precise debridement of necrotic or metastatic tissue, targeted anterior reconstruction, and minimal soft-tissue disruption under direct visualization. Such developments may broaden the applicability of intrabody cage augmentation across fragile or neoplastic spinal conditions, establishing it as a versatile tool in minimally invasive spine surgery.

A critical question remains whether a rigid cage is superior to impacted bone grafting or cement augmentation. While bone chips or allografts avoid the risks associated with a large foreign body, they may lack the immediate structural integrity required to restore vertebral height in cases of severe collapse. Cages provide immediate mechanical column support; however, they carry risks such as cage migration or retropulsion into the spinal canal. Currently, there is a significant gap in the literature regarding direct comparisons between cage augmentation and bone grafting alone, and this warrants future prospective investigation. Furthermore, it is crucial to acknowledge the methodological limitations of the existing literature. As summarized in Table 1, the current body of evidence is primarily restricted to Level IV retrospective case series with limited sample sizes and a distinct lack of randomized control groups. These constraints inherently introduce significant selection bias, making it difficult to draw definitive, broad-scale conclusions regarding the superiority of intrabody cage augmentation over standard practices. Consequently, the long-term durability and safety profile cannot yet be definitively established. To address these limitations, further high-quality prospective investigations and comparative trials are essential to validate the definitive clinical role and long-term efficacy of this technique.

## 11. Conclusions

Intrabody cage insertion represents a minimally invasive surgical adaptation that may serve as an emerging potential surgical option for the management of Kümmell disease and fragile osteoporotic burst fractures. By utilizing the IVC for the off-label placement of interbody devices, this approach restores vertebral height, corrects local kyphotic deformity, and reduces implant stress, avoiding the morbidity associated with anterior corpectomy. Nevertheless, the current evidence supporting this technique remains preliminary. Future high-quality, large-scale prospective comparative studies are essential to establish definitive safety profiles, long-term durability, and standardized treatment guidelines.

## Figures and Tables

**Table 1:** Representative Studies on Intrabody Cage Insertion for Osteoporotic Burst Fracture and Kümmell Disease.

Year	First Author	Title/Journal	Study Type and Key Findings
2007	Li KC	Another option to treat Kümmell’s disease with cord compression (*Eur Spine J.* 2007) [7]	Retrospective series (*n* = 21). Used transpedicle body augmenter (TpBA, titanium spacer) with manual reduction. Demonstrated effective kyphosis correction, vertebral height restoration, and 81% good clinical outcomes.
2018	Sung Ho Lee	Transpedicular Intrabody Cage Insertion with Posterior Spine Stabilization in Kümmell Disease: Report of 2 Cases (*World Neurosurg*. 2018) [8]	Case report (*n* = 2). Demonstrated feasibility of intrabody cage insertion with posterolateral fusion. Both patients achieved pain relief, maintained vertebral body height, and achieved successful fusion.
2020	Jeong-ik Lee & Kwang-Sup Song	Transpedicular Intravertebral Cage Augmentation in a Patient with Neurologic Deficits After Severely Collapsed Kümmell Disease: Minimum 2-Year Follow-Up (*World Neurosurg.* 2020) [9]	Retrospective series (*n* = 10). Posterior-only intravertebral cage augmentation for severely collapsed vertebrae. Achieved significant correction of local kyphosis, ODI improvement, and restoration of ambulation.
2020	Changjun Chen	Intravertebral insertion of interbody fusion cage via transpedicular approach for stage III Kümmell disease: Technical note (Preprint. 2020) [10]	Technical note. Modified transpedicular interbody cage insertion with posterior stabilization. Reported pain relief, anterior height restoration, and kyphosis correction without major complications.
2020	Lin Chen	Minimally Invasive Decompression and Intracorporeal Bone Grafting Combined with Temporary Percutaneous Short-Segment Pedicle Screw Fixation for Treatment of Thoracolumbar Burst Fracture with Neurological Deficits (*World Neurosurg*. 2020) [11]	Retrospective case series (*n* = 18) Combined minimally invasive decompression, intracorporeal bone grafting, and temporary short-segment fixation. Achieved kyphosis correction, vertebral height restoration, neurological improvement, and solid fusion. Proposed as a less invasive alternative to open corpectomy.
2022	Hyun-Jin Park	Percutaneous transpedicular intracorporeal cage grafting for Kümmell disease (*Acta Neurochir.* 2022) [12]	Technical note. Introduced PTICG with short-segment fixation. Provided minimally invasive anterior column reconstruction while preserving posterior elements.
2024	Kwang-Sup Song	Transpedicular Intravertebral Cage Augmentation Using Expandable Cage in Kümmell Disease: Technical Note and Case Series (*World Neurosurg*. 2024) [13]	Case series (*n* = 6). Expandable cage via TPICA. Achieved improved vertebral height restoration and correction angle; all patients regained independent ambulation.
2024	Junseok Bae	Minimally Invasive Surgery Transpedicular Intrabody Cage Technique (MISTIC) for the Management of Kümmell Disease (*Int J Spine Surg*. 2024) [14]	Retrospective series (*n* = 20). Employed MISTIC. Achieved significant kyphosis correction, vertebral height restoration, and 100% fusion rate at 12 months.

Abbreviations: TpBA, Transpedicle body augmenter; PTICG, Percutaneous transpedicular intracorporeal cage grafting; TPICA, Transpedicular intravertebral cage augmentation; MISTIC, Minimally Invasive Surgery Transpedicular Intrabody Cage Technique; ODI, Oswestry Disability Index.

**Table 2:** Treatment Strategies for Osteoporotic Burst Fracture and Kümmell Disease.

Technique	Typical Indications	Advantages	Limitations	Key References
Cement augmentation (Vertebroplasty/Kyphoplasty)	Stage I or early Stage II KD; painful OVCF without major collapse or canal compromise	Minimally invasive; rapid pain relief; short operative time	Ineffective for advanced collapse or instability; cement leakage risk; increased incidence of adjacent fractures	Ruiz Santiago et al. (2014) [28]; Ha et al. (2010) [29]
Posterior screw fixation	Severe instability; multi-level involvement; poor bone quality	Provides more robust stabilization and kyphosis correction rather than cement augmentation; technically less demanding	Risk of implant failure in osteoporosis; intermediate surgical morbidity	Li et al. (2014) [18]; Liao et al. (2019) [21]
Anterior corpectomy with posterior screw fixation	Stage III KD with severe canal compromise and disruption of the endplate or adjacent disc	Direct ventral decompression; solid anterior column reconstruction	Technically demanding; risk of implant failure in osteoporosis; high blood loss; cardiopulmonary risk in frail older adults	Li et al. (2007) [7]; Chen et al. (2023) [10]
Intrabody cage augmentation (Transpedicular approach)	Stage II KD (without canal compromise; no laminectomy) Stage III KD (with canal compromise; requires laminectomy) (Must have preserved endplates)	Restores vertebral height and sagittal alignment; avoids morbidity associated with anterior corpectomy; preserves adjacent discs	Technically straightforward; pedicle diameter limitations; risk of cage subsidence; limited long-term data	Lee et al. (2018) [8]; Lee & Song (2020) [9]; Song et al. (2024) [13]; Bae et al. (2024) [14]

Abbreviations: KD, Kümmell disease; OVCF, osteoporotic vertebral compression fracture.

## Data Availability

The raw data supporting the conclusions of this article will be made available by the authors on request.
